# Going vertical: examining the rise and impact of contemporary Russian sports cinema

**DOI:** 10.3389/fspor.2023.1250961

**Published:** 2023-11-06

**Authors:** Seán Crosson

**Affiliations:** Huston School of Film & Digital Media, University of Galway, Galway, Ireland

**Keywords:** sport cinema, Russian cinema, European cinema, sport, film

## Abstract

**Introduction:**

While Hollywood has been central to the definition and popularisation of the genre internationally, sport cinema also has a significant presence in national cinemas across the world, though less research has been undertaken to date of the place of sport within European cinema and its growing importance in this context. This article will introduce a new database and online research platform (Sport in European Cinema, sportandfilm.eu) recently made publicly available which enables a deeper consideration of the significance of sport in European cinema, including its increasing importance in the Russian context. Indeed the third most commercially successful indigenous film ever released (as of 2023) at the Russian box office is a sports film, *Движение вверх* (*Going Vertical* (AKA *Three Seconds*) (2017).

**Methods:**

This article adopts a mixed-methods approach, combining quantitative and qualitative methods to examine the place of sport in European cinema, including a case study of contemporary Russian sport cinema and its connection with broader political and ideological messaging.

**Results:**

Sport cinema has become an increasingly important part of European cinema, both in terms of number of films produced and their impact on European society, as evident in the case study provided of Russian sport cinema.

**Conclusion:**

This paper introduces a new research database dedicated to European Sport Cinema and identifies the increasing importance of sport cinema in the European context. It includes a case study of contemporary Russian sport cinema and identifies its role in articulating and affirming existing power structures and ideological hegemony within Russian society, as evident within *Going Vertical* and further recent Russian sport films in a particularly challenging period of national and international instability and contestation.

## 1. Introduction

Sports films have achieved considerable commercial success and critical acclaim in the Anglophone world. On June 17, 2008, in a CBS television special, the American Film Institute included the Sports film genre as one of the ten most important in American film history (1). While Hollywood has been central to the definition and popularisation of the genre internationally, sport cinema also has a significant presence in national cinemas across the world, though less research has been undertaken to date of the place of sport within European cinema and its growing importance in this context. This article will introduce a new database and online research platform (Sport in European Cinema, sportandfilm.eu) recently made publicly available which enables a deeper consideration of the significance of sport in European cinema, identifying salient aspects of the genre and its revealing engagement with European society and culture. In particular, drawing from the database findings and related research this article will examine the growing popularity and impact of sport cinema in the Russian context, above all from the early 2000s onwards. Indeed the third most commercially successful indigenous film ever released (as of 2023) at the Russian box office is a sports film. Russian sports films have had limited impact outside of their home market; the only other major market for the film concerned—*Движение вверх* (*Going Vertical* (AKA *Three Seconds*) (Anton Megerdichev, 2017)—was in China, where it took $13,287,714 of its total box office of $108,613,501, becoming the most commercially successful Russian film ever released in that country (2).^1^ Such has the film's impact being in Russia that it became an important part of the domestic discourse concerning Russia's response to international criticism for both sporting and military actions over the past ten years. Indeed, this article will contend that *Going Vertical* also contributed to the grooming of a domestic audience for subsequent military actions (particularly in Ukraine), a process to which Russian sport cinema has made an important contribution. As cultural studies scholars have long argued, film plays a critical role as a mediator of social relations and in affirming hegemonic ideologies through the naturalization of cultural and societal norms (3–5). Mindful of this critical discourse, and through textual and contextual analysis, this paper examines the articulation and affirmation of existing power structures and ideological hegemony within Russian society as evident within *Going Vertical* and further recent Russian sport films in a particularly challenging period of national and international instability and contestation.

## 2. Methods

While considerable research has been undertaken on the history of sport (including basketball) in Europe and internationally [see for example (6–10)] and on the depiction of sports in film and other visual media [see (11–19)], most studies to date have focused on the American context. Studies of European sports films have tended to focus on individual case studies of exemplary films—e.g., *Olympia* (Germany 1938);^2^ *Chariots of Fire* (UK 1981);^3^ *Les Triplettes de Belleville* (The Triplets of Belleville, France 2003))^4^—or the development of sport cinema within specific national contexts—e.g., Jones's (20) work on the British sports film; Romaguera i Ramio (21), Rodríguez Díaz (22), and Ashton (23) on the Spanish sports film; Cunningham (24) and Fodor (25) on Hungarian football films; McDougall (26) on East German football films; or the work I have done on Gaelic games on film in Ireland (27).

However, to consider sport cinema in the broader European context, it seemed necessary for me to identify the extent and nature of this cinema as it has developed historically across Europe as a whole and what salient features this might reveal regarding sport cinema in specific national and regional contexts. While film studies has been dominated by qualitative approaches (including in my own research to date), quantitative work on genre has been evident for some time. An early example was John B. Kuiper's mid-1960s study of American civil war films where he noted, in words very relevant also to sport cinema, that while many of the relevant films have justifiably been ignored by critics due to their questionable quality, nonetheless

The film historian … ought to be aware of all the products of a given genre of film production. To an historian, a difference in quantity ought to be critical because in the spectator- oriented, popular art world of the film, the number of films made during a given decade or year can be indicative of the opinions, attitudes, and conceptions of both the film makers and the audiences of the period (28)

With regard to sport cinema in particular, apart from themed filmographies on the genre by Wallenfeldt and Williams respectively, one in-depth quantitative survey has been undertaken, though (perhaps understandably given that the majority of sports films have emanated from this source) in the American context. Pearson, Curtis, Haney, and Zhang, in their article “Sport Films: Social Dimensions Over Time, 1930–1995”, identified 590 American films in that period, and examined various trends apparent, including “sports depicted in films and their shifts over time, film content and theme, and social and cultural relevance” (29). This article has been particularly useful for the purposes of informing my own quantitative survey, and the methodology applied to date, including with regard to the various tables and graphs used to illustrate findings in this paper.

The research towards the compilation of the Sport in European Cinema database was not without its challenges including the lack of an entirely comprehensive database of European cinema; those databases that do exist—including The European Film Directory—do not currently provide the facility to search by sport or sport genre. While recognising the importance of non-fiction sport films in Europe (both as influential film texts and important historical records of specific sports), due to the sheer number of relevant films involved, I have focused to date in our database on fictional and theatrically released works produced or co-produced by or with a European company that feature sport, sports people, or followers of sport prominently and in which sport is an important aspect of the plot development or motivation. However, the challenge in this exercise was not just deciding what films qualify as sport films (and how we define that term) but equally the linguistic challenges we encounter in attempting to undertake a pan-European study of a popular cinema genre. For our purposes, we began with perhaps the most well-known and comprehensive database of international cinema, the Internet Movie Database (IMDb). In deciding to start here, I was entirely conscious of the limitations of this resource, including the gaps that exist regarding international cinema but also the issues with how films are classified there. What IMDb does offer, however, is the possibility to identify sport, feature film, and country, as categories allowing us to examine what is classified in this way and then to extrapolate from there. In total I found relevant sport films listed in 37 European countries. Certainly this is an imperfect measure and one that needs to be interrogated at each point (as I have been doing as I compiled my database) and has been supplemented by relevant studies of the genre and national and international online databases where available. It was this broader research that identified noteworthy patterns in the development of sport cinema in specific European contexts, leading to the case study of the Russian experience developed below.

## 3. Results and discussion

**Figure 1:**
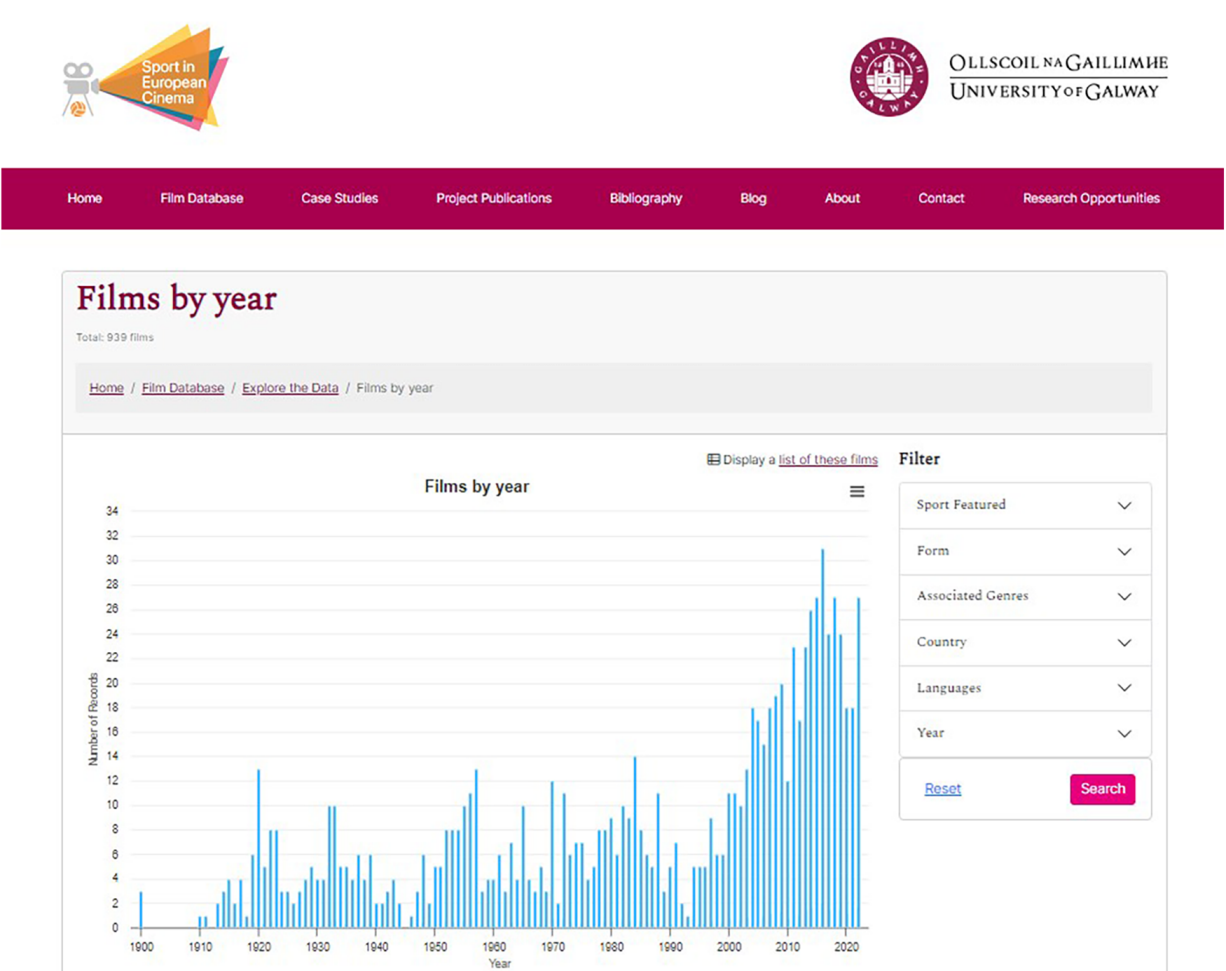
Growth of European sport cinema: 1910–2022.

The first striking feature from the research we undertook and the final database produced was the sheer number of relevant films (even focusing only on theatrically released fiction work)—currently 939 between 1910 (when the first fiction production, the British made *Rogues of the Turf* was made) and 2023, with the following graph (Figure 1) indicating the growth of production in that period.

Examining the distribution of sports across films over the past century or more indicates a number of further salient features. Firstly, association football is by far the most popular sport featured, depicted in almost one quarter of all films produced followed by boxing, horseracing, and athletics.

Secondly, we can identify differing sports coming to prominence in differing periods, reflecting larger social changes of the times concerned. For example, while horseracing is modestly represented overall (featuring in approximately 9% of films), this is concentrated in the UK and in particular in the 1920s when around 50% of all films featuring horseracing were released. This was a period in which, as Mike Huggins has noted, “Horseracing has a powerful claim to be Britain's leading interwar sport” (30) and this is reflected in the large number of films produced in the period featuring horseracing.

Further patterns evident from our analysis is the increasing number of sports featured in film from the 1970s onwards, more than doubling from 25 up to the 1960s to over 60 sports represented by 2022, while the same period has also seen a very significant increase in the number of sports films produced, as evident in Figure 1. Our data also allows us to assess what the dominant associated genres are, with drama dominating, though comedy also features prominently.

But how does this relate to individual countries? Our data indicates that by far the most prolific country to produce or co-produce sport cinema is the UK, followed by Germany, France, the Soviet Union (and subsequently Russian Federation), Spain and Italy. The high number of British produced or co-produced sport films reflects on one level the prominence of sport in the UK (particularly football, evident in the high number of football films produced there). However, the fact that the UK also shares a language and strong cultural ties with the US (the producer of most sport films) has also contributed to the large number of sport films produced in the country. This is evident in the high number of US/UK co-produced sports films, with 13% of UK produced sports films having an American co-producer.

**Figure 2:**
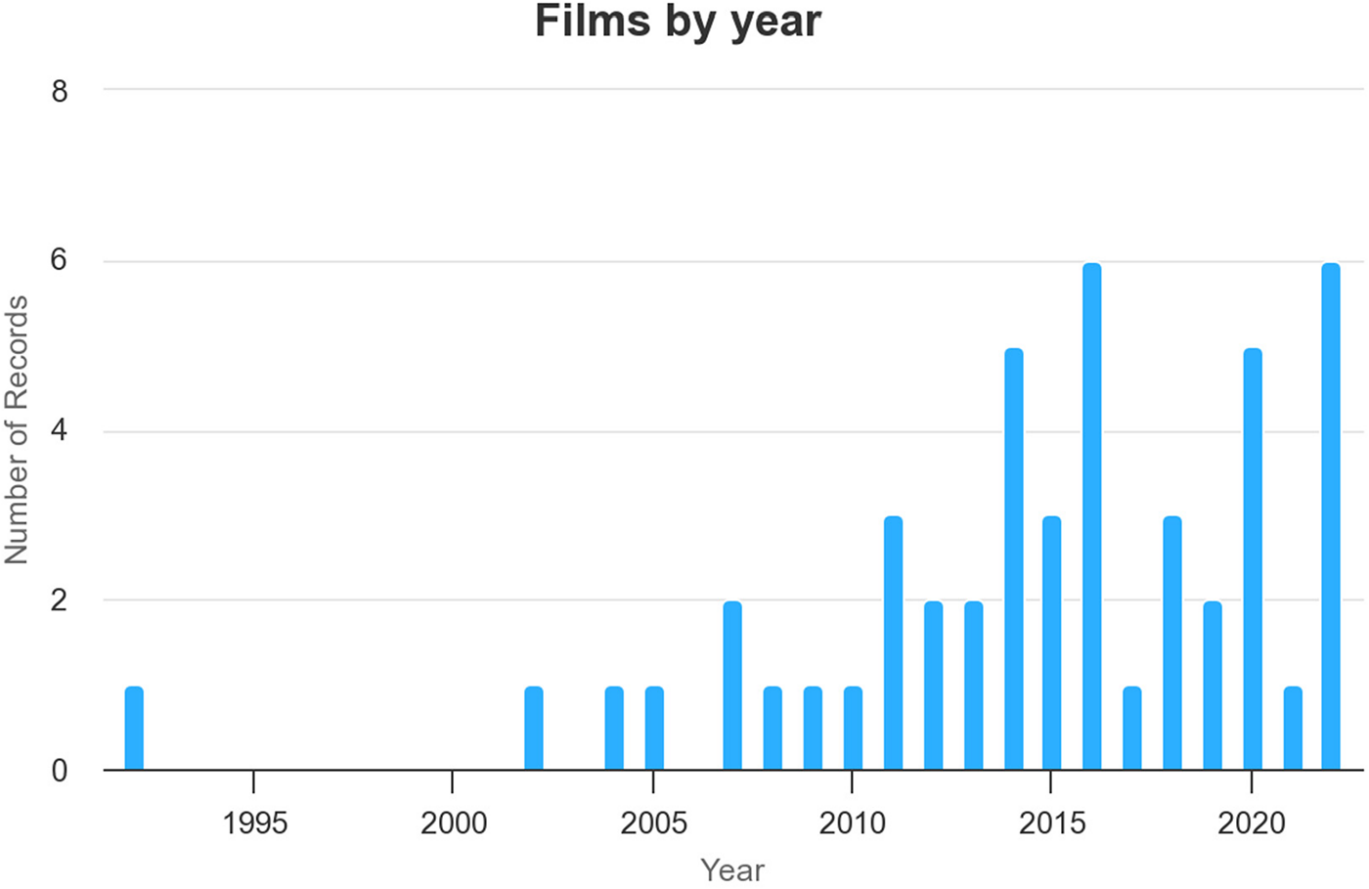
Chart depicting growth of production of Russian sports cinema 1992–2022.

During the research I was struck by the increasing presence of the sport cinema genre in the Russian context over the past twenty years (see Figure 2), a period that coincides with the presidency, prime ministership, and presidency again of Vladimir Putin. The distinctiveness and importance of sport in the Soviet Union and subsequently Russian context, and its connection with broader political priorities and international relations has been the subject of considerable research, including in the work of Riordan (31), Gounot (32) and Sylvain Dufraisse's (33) recent consideration of the insight sport provides into the period of the “Cold War” as an important space for the expression of antagonism between the two superpowers. The increased presence of sport in contemporary Russian cinema therefore builds on important historical precedents, but also parallels a larger national project associated with the regime of Putin (himself a keen sportsman) whereby sport has been employed as a key vehicle for the promotion of nationalism and patriotism in Russia today. As Richard Arnold [in line with many other recent commentators (34–37)] has noted:

sports policy is a central part of the Russian government's strategy to promote nationalism and … it is consciously evoking the Soviet legacy as a model in doing so. There are numerous reasons to suggest that the Russian state is well positioned to embrace sports as an important means of nation-building. First, it is a relatively young country searching for a national idea and identity in the aftermath of the Cold War… Second, like Germany, Russia largely lacks a past that is truly non-divisive. Many parts of the Soviet legacy are profoundly alienating to certain segments of Russian society, and so sporting greatness, offensive to none, is a valuable attribute. Third, Russia's particular ethnic mosaic means that monoethnic nationalism is not a viable option (Kolsto et al. 2016; Putin 2012), so the regime has to find other ways of emphasizing civic nationalism as part of the “national idea.” Promoting popular culture, including sports, is one way to achieve this goal (38)

However, despite Russia's success in winning the bidding processes to host both the Sochi winter Olympics (2014) and the subsequent FIFA world cup (2018), both events were greatly tarnished from an international perspective firstly by the annexation of Crimea (a process which began during the Sochi Olympics) and subsequently by the revelations of a state-sponsored doping regime that led to a ban been imposed by the International Association of Athletics Federations [IAAF] on Russian athletes competing in international competitions. Film (which is heavily state funded in the country) has provided an important platform for Russia to articulate its response (if principally to a domestic audience) to increasing international criticism.

While it is understandable in the context of an increasing focus on sport more generally in Russia that sport should feature more prominently in the country's cinema output, the sports featured and the overall focus of these films is particularly interesting. Firstly, there is an evident emphasis apparent on sports dependent on physical prowess (predominantly in association with male athletes)—in particular martial arts—with over one third of the Russian sports films produced since 1992 focused on boxing, MMA or wrestling. This focus on physicality is also evident across many of the other films depicting team sports—including most prominently football, ice-hockey and basketball. Secondly, there is a recurring focus on patriotism (often located with regard to the Soviet past), evident in some of the most commercially successful of the sport films releases in Russian. This includes *МАТЧ* (*Match*) (Andrey Malyukov 2012), concerning the so-called WWII “death match” between Kiev's FC Start (composed of workers from a local bread factory, but many of whom had played with local professional team Dynamo before the war) and German team Flakelf (comprising Luftwaffe or anti-aircraft personnel). While the prevailing narrative suggested that the Start players were threatened with being killed by the Germans if they won, and many were executed subsequently for doing precisely that (5-3), this narrative was largely based on soviet propaganda (39). The contemporary spin in the Russian 2012 release is that all the Nazi collaborators speak Ukrainian while the Russian speakers are depicted much more positively (including members of the Start team) (40), a contrivance that anticipates and connects with the contemporary justification provided by Vladimir Putin and his regime for the full-scale invasion of Ukraine on February 24 2022 (41).

Further sport cinema commercial successes include *Легенда №17* (*Legend No. 17*) (Nikolay Lebedev, 2013) concerning the rise to fame of the legendary Soviet ice-hockey player Valeri Kharlamov [a film that is apparently a personal favourite of Vladimir Putin (42)] and *Chempiony* (Champions) (Artyom Aksenenko, Dmitriy Dyuzhev, & Emil Nikogosyan 2014), five profiles of actual Russian Olympic athletes emphasising (according to the film's publicity material) victories earned through “hard work, respect and belief in yourself, in your family and to your country.”^5^

However the sports film that has had the greatest impact on the Russian box office [to date the third most commercially successful indigenous film ever released (as of 2023) taking $95,225,622 in Russia alone]^6^ is the 2017 basketball-themed production *Dvizhenie vverkh* (*Going Vertical*) (Anton Megerdichev 2017). Interestingly, the only other major market for the film was China where the production became “the highest grossing Russian film in China, with an 85 million yuan ($12.3 million) gross” (2). Such has the film's impact being in Russia that it became an important part of the domestic discourse concerning Russia's response to international criticism for both sporting and military actions over the past ten years with at least one media outlet even suggesting that the film's success may have contributed to ensuring the reelection of Vladimir Putin (43).

The film's subject is a very rare and unexpected victory for the Soviet Union over the all-conquering United States in the men's basketball final at the 1972 Munich Olympic Games. From the addition of basketball to the Summer Olympics as a sport at the 1936 Berlin Olympics, the USA has dominated the competition, winning the gold medal at every subsequent tournament without losing a single game prior to the 1972 Munich Olympics (44). American dominance is perhaps unsurprising: basketball had its origins in the United States where it was invented in 1891 by Canadian physical education instructor James Naismith (then based at the International YMCA Training School in Springfield, Massachusetts) and the United States is also the location of the most popular and commercially successful basketball league featuring the top players in the world, the National Basketball Association (NBA). As a sport, it has become established as an important popular culture representation of American identity and ideology (particularly in its association with the American dream), and during the Cold War provided a significant international symbol of American cultural ascendency internationally (45, 46). As such, the defeat of the United States in the basketball Olympic final in 1972 provided very significant cultural capital, both domestically and internationally, for the Soviet Union at the height of the Cold War.

The timing of the release of *Going Vertical* was also highly significant: it was three weeks after the decision by the International Olympic Committee (IOC) to bar the Russian team from the 2018 Winter Olympics in South Korea (following the discovery of a state-sponsored doping regime by Russia) and three months before the Russian Presidential election. The film would become bound up with both these events in the discourse that followed its release. After a closed screening of the film in Moscow in early December 2017, Russian news anchor for the main state channel Rossiya 1 (and head of the official Russian government-owned international news agency Rossiya Segodnya) Dmitry Kiselyov, remarked that many in the audience “left the theater in tears but also as if they were flying” (47). As the *Radio Free Europe/Radio Liberty (RFE/RL)* report of the screening continued,

Kiselyov, known for his colorful anti-American diatribes on his weekly news analysis program, predicted it will become “the essential film of the entire New Year holidays.” The timing of the film's release has already drawn it into the discussion surrounding the decision last week by the International Olympic Committee (IOC) to bar the Russian team from the Winter Olympics in Pyeongchang, South Korea, in February. Kiselyov's segment on the film aired two days before the IOC's December 5 announcement of the punitive measures … “Of course, the doping scandal surrounding our national team is a difficult, unfair, and painful situation. But when has it ever been easy for our athletes?” Kiselyov said in his introduction to the report, which included a sarcastic swipe at the World Anti-Doping Agency (WADA) (47).^7^

Such was the popularity of *Going Vertical* that it was estimated that the film in its first 3 weeks on release was “watched by over 9 million people or approximately one in 12 registered voters” (43).

*Going Vertical* emphasises repeatedly patriotism, which is perhaps to be expected given the allegiances of those behind the work [as well as the fact that it was largely state-funded (43)]. The film was produced by Nikita Mikhalkov, head of the Russian Cinematographers' Union, and chairman of one of Russia's most successful production houses, Three T productions. He is also one of Putin's strongest supporters; he produced a television program for Putin's 55th birthday, co-signed an open letter asking Putin not to step down after the expiry of his second term in office in 2008, and subsequently received from president Putin on 10 December 2015 the 1st Degree Order of Merit for the Fatherland—the highest civilian honour in Russia (48). At *Going Vertical*'s premiere at the October Cinema in Moscow on December 22nd 2017, Mikhalkov told the audience that the movie was designed to dilute what he described as

Western hostility toward Russia as shown by the banning of its Olympic and Para-Olympian teams. It is a counterweight to what is happening to us with sanctions, double standards, untruth, fakes and lies. It's a film that like an arrow pierces all of this (47).

The lead actor in *Going Vertical,* Vladimir Mashkov (who plays Soviet coach Vladimir Garanzhin), also has a close connection with the current Russian president—Mashkov formally nominated [as a prominent member of the All-Russia People's Front (ONF)] Putin for re-election on December 26, 2017 ahead of the March 18, 2018 vote. Indeed, as one of Russia's most prominent film actors, he was featured heavily in the Russian media at the time of that nomination (49).

**Figure 3:**
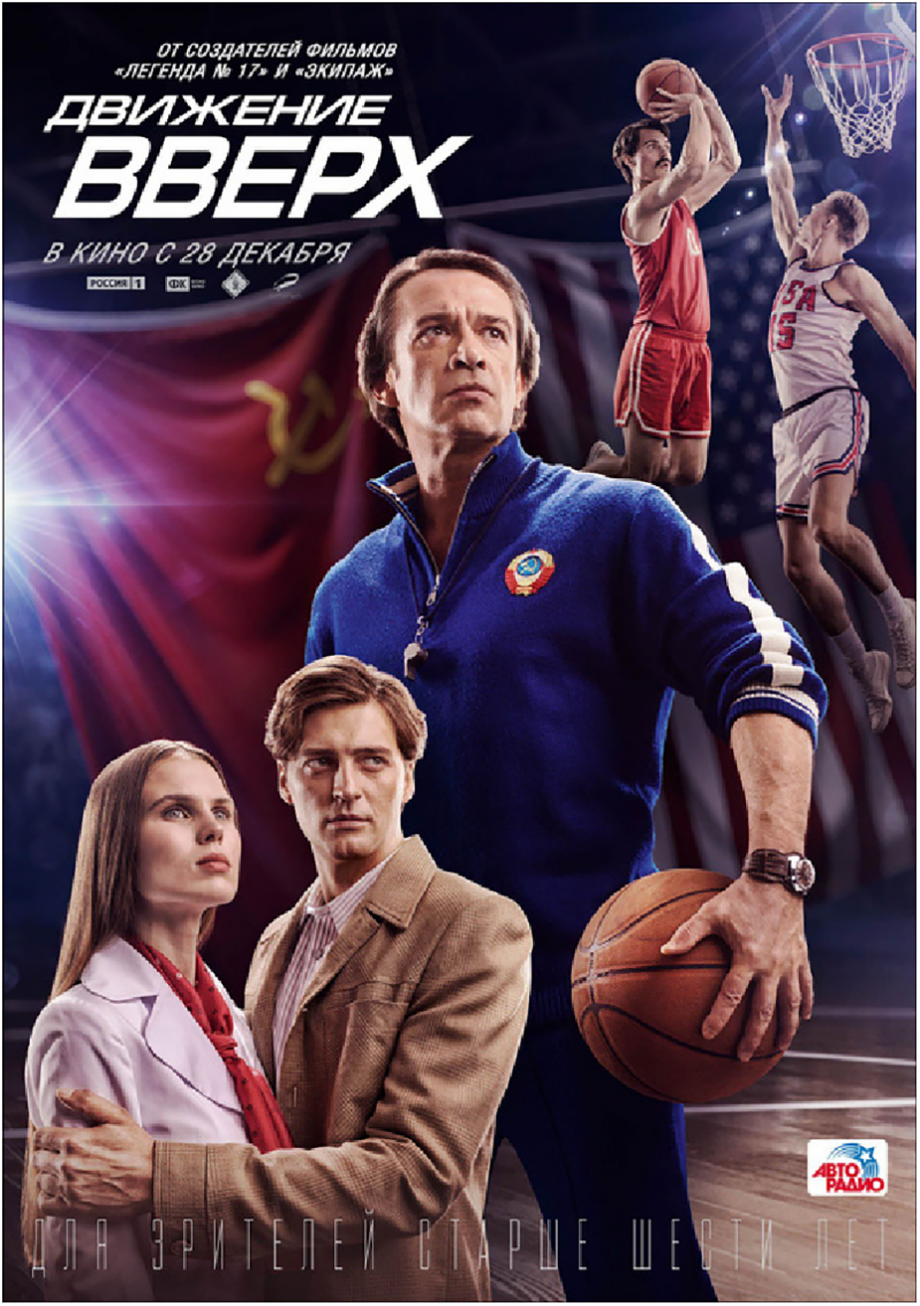
Poster for *Движение вверх* (*Going Vertical*) (Anton Megerdichev 2017).

**Figure 4:**
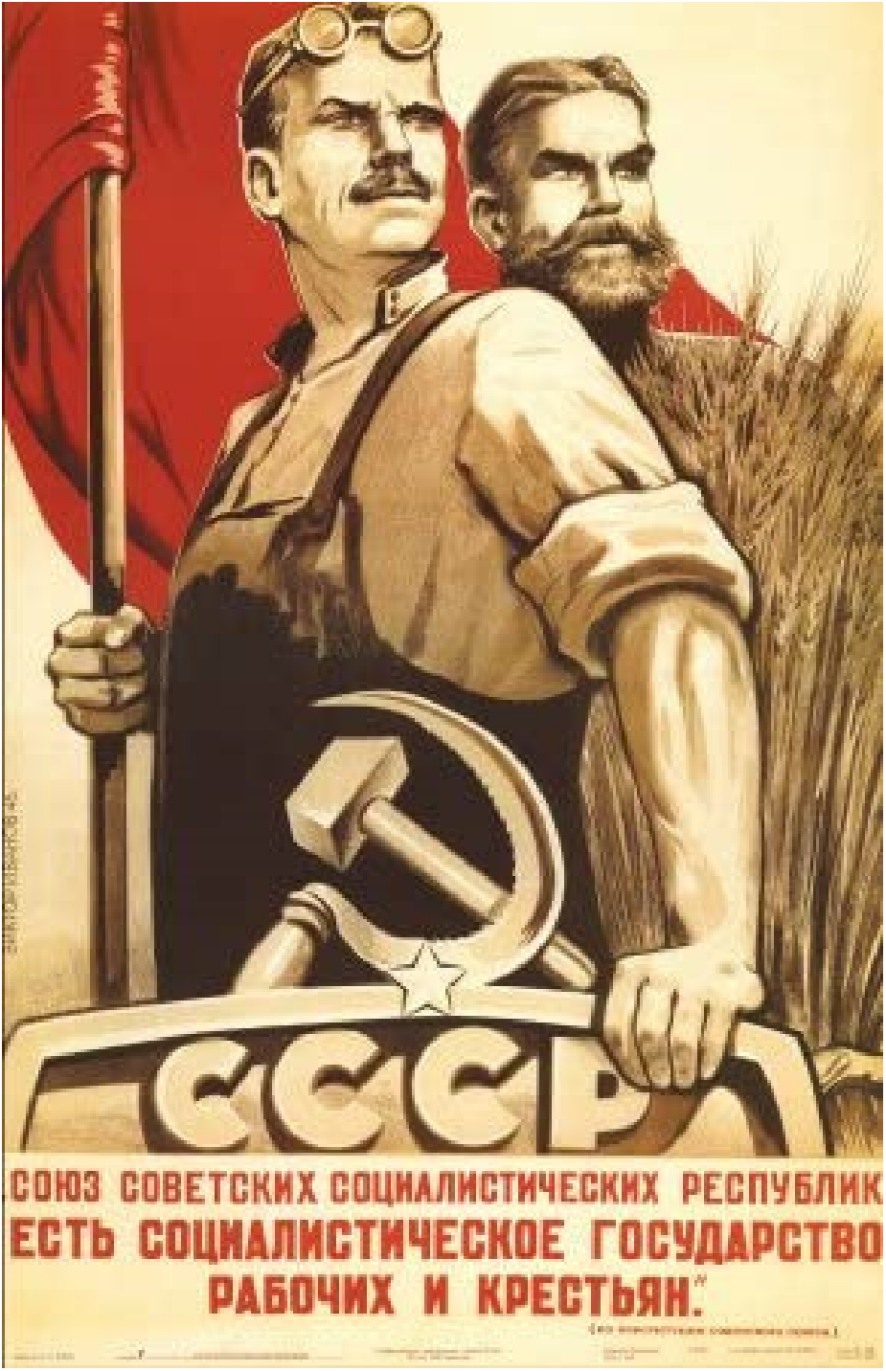
Classic soviet propaganda poster from 1945 (Viktor Semenovich Ivanov) source: alamy.com^8^.

The film's poster (see Figure 3), designed by Russian art designers Anna Uyutnova, and Anna Nikolaeva under the direction of Egor Sheremetiev for the Central Partnership distribution company (50), is itself fascinating and distinctive in its rendering of sporting figures. Indeed, in its depiction of central characters in the film standing heroically and gazing into the distance with the Soviet flag draped behind, it resembles remarkably the classic Soviet poster depicted in Figure 4 and produced by seminal poster artist Viktor Semenovich Ivanov in 1945 depicting an idealized image of workers. This poster was typical of the propaganda spread by the USSR's communist government in the mid-twentieth century: the translated text reads “The USSR is the socialist state for factory workers and peasants.” Such was the regard with which Ivanov's work was held he was awarded “the Stalin Prize for his wartime contributions in 1946 and 1949 and the title Honored Worker of Arts of the Russian Soviet Federative Socialist Republic in 1955” (51).

A fascinating aspect evident when comparing these two posters is what is held in the hand by the central figure in each. Whereas the principal character in Ivanov's poster is clutching a device emblazoned with the letters of the USSR, this is replaced in coach Garanzhin's hand by a basketball, suggesting that sport can perform the role undertaken previously by the Soviet Union as a political entity to bring together the diverse cultures and ethnicities today inhabiting the Russian Federation. It is this theme to which I will now turn in considering the film.

*Going Vertical's* patriotism is evident particularly in its focus on the efforts of the Soviet basketball team to overcome the seemingly unconquerable Americans, but also in how the film transcends through sport the divisions between members of the Soviet team. The film contrasts, for example, in its depictions of the climactic Olympic final game against the United States the noble and heroic acts of Soviet players with the underhand and violent actions of their American competitors. Indeed, earlier in the film during a game against a US College basketball team, it is strongly suggested that the use of banned substances by the Soviet players was inspired by actions witnessed by Soviet players of an American basketball player already benefiting from these illegal methods. The irony of course is that in many respects the film is hugely indebted to American culture and society. Firstly, the film follows the pattern (in every respect) of the most successful American sports films, including the overall underdog trajectory that sees a team rife with internal conflict coming together to achieve victory; the conflicted and tortured coach figure trying to bring new ideas into the team (Vladimir Garanzhin, who is also attempting to leave the USSR to get medical treatment for his disabled son); the extended montage sequences that follow the Soviet team's development of both individual skills and team cohesion; and the climatic final game that eventually brings victory (after repeated defeats earlier in the film) for the Soviet team. Furthermore, the principle contribution of the new coach who joins the team at the outset is bringing what he calls “American methods” to their play, ostensibly a faster moving and passing approach to the game (but perhaps also the use of performance enhancing substances!); the coach also insists that his players have the opportunity to play against American teams in preparing for the Olympics and is given permission to do so by his superiors.

*Going Vertical* also speaks repeatedly and directly to the audience regarding its national identity (read Russianness), and defines this in broad and patriotic sporting terms. One of the major conflicts in the team concerns tensions between players from differing parts of the Soviet Union, including Lithuania (the home country of the team's captain Modestas Paulauskas), Georgia (Zurab Sakandelidze) and Russia itself. These are resolved in the film through an appeal to sport, invoking loyalty to one's teammates (and the team as a whole) as a means to overcome these ethnic differences, an appeal that shares strong parallels with Putin's own policies in recent years: Paulauskas in particular repeatedly asserts his distinctive Lithuanian identity, and threatens to leave the team to play for better money elsewhere in Europe: however, ultimately he is convinced that his real family is the sporting teammates he has made in the team itself and he returns to play in the Olympic final.

It may come as no surprise to learn that, with the exception of the climactic events in the film, the narrative strays considerably from historical fact. With regard to Paulauskas, as noted by Sidorchik and others, there is no historical evidence to indicate that Paulaustus was indeed a dissident who had plans to defect (52). What we find in this contrivance is but one of a series of aspects of the film that are more concerned with responding to contemporary anxieties than historical accuracy. There is in this aspect of the film a clear messaging to former Soviet Republics (including arguably Ukraine) that their best interests lie in orientating towards Russia rather than the West.

## 4. Conclusion

This article has shared some initial findings, including a case-study of Russian sport cinema, from the recently made available Sport in European Cinema database (sportandfilm.eu). The database reveals the perhaps underestimated importance of sport cinema in the European context, particularly in terms of its impact and popularity in specific European contexts. Sport cinema provides a unique and valuable moving image resource for those seeking to understand the development of sport in Europe since the early twentieth century, and as with all popular culture, its very popularity among audiences, and the ways in which specific film texts may have informed norms, values and understandings of topics and themes featured, requires deeper consideration and further research. Perhaps unsurprisingly, our database reveals association football as by far the most popular sport featured in European sport cinema. However, it also reveals the coming to prominence of specific sports in different eras, reflecting larger social changes; the increased production of sport films since the 1970s and significant expansion of the range of sports featured; and the dominance of drama and to a lesser extent comedy as the principal associated genres. With regard to national production, the UK is by far the most significant producer (in terms of output) of sport cinema, though the database also reveals the increasing importance of this genre across a range of national contexts, including in the Russian Federation since the early 2000s. In our case-study of Russia, we identify significant parallels between this development and broader sport-focused initiatives led by President Vladimir Putin. In common with these initiatives, Russian sport cinema also stresses patriotism while revealing anxieties regarding the threat posed by the West, particularly the United States. One of the most popular indigenous films ever released in Russia, *Going Vertical*, epitomises a number of features found across European sport cinema today but particularly in Russia: these films (above all in their most popular manifestations) are very influenced by the dominant American model of the genre that foregrounds what we might call the underdog trajectory; these films are also working to overcome (using the seductive, appealing and emotionally charged sporting context depicted) contemporary tensions within specific European societies. While recognizing and acknowledging the increasing importance of co-production arrangements in recent decades—facilitated by EU and Council of Europe funding initiatives—the overriding focus of this genre in Europe remains on national rather than transnational concerns. As such, one might view the sports film genre in Europe as more often a barometer of and significant contributor to nationalist feeling—frequently sport is invoked (or one could contend employed) in film to accentuate nationalist feelings, particularly at moments of crisis or perceived threat to hegemonic national culture. Significantly, *Going Vertical* was re-released on March 5th 2022 in Russia (a little over one week after the full-scale invasion of Ukraine on February 24) when it enjoyed its biggest success at the Russian box office, taking $48,728,504 of its total domestic gross of $95,225,622. In the words of Eric Hobsbawm (speaking of football rather than basketball but nonetheless still relevant) “*The imagined community of millions seem more real as a team of eleven*” (52) and film has provided a powerful platform in Russia and the broader European context to realise this possibility.

## Data Availability

The datasets presented in this study can be found in online repositories. The names of the repository/repositories and accession number(s) can be found below: https://sportandfilm.eu/.

## Footnotes

^1^Box office figures available on IMDb at https://www.imdb.com/title/tt5987402/ and on Box Office Mojo at https://www.boxofficemojo.com/title/tt5987402/?ref_=bo_se_r_1

^2^See for example Taylor *Downing (1992)*, *Olympia*. London: BFI Publishing; Hannah B Schaub, (2003) *Riefenstahls Olympia: Körperideale-ethischeVerantwortungoderFreiheit des Künstlers?*München: Wilhelm Fink Verlag; and María Graciela Rodríguez, (2003) “Behind Leni’s Outlook: A Perspective On The Film Olympia (1938)” *International Review For The Sociology Of Sport* 38.1: 109–116.

^3^See for example Steve Neale, (1982) “Chariots of Fire', Images of Men” *Screen* 23.3–4: 47–53; Martyn Bowden, (1994)“Jerusalem, Dover Beach, and Kings Cross: Imagined Places as Metaphors of the British Class Struggle in *Chariots of Fire* and *The Loneliness of the Long-Distance Runner*”. In Aitken, Stuart C. &Zonn, Leo E. (Eds.) *Place, Power, Situation and Spectacle: Geography of Film* Lanham, MD; London: Rowman & Littlefield Publishers. pp. 69–100; and Ellis Cashmore, (2008) “Chariots of Fire: bigotry, manhood and moral certitude in an age of individualism”, *Sport in Society*, 11:2–3: 159–173.

^4^See for instance Annie Jouan-Westlund, (2006) “L'imaginaire populaire Franco-Américain dans l*es Triplettes De Belleville*”*The French Review* 79.6: 1195-1205; F. Place-Verghnes, (2007)“Douce France? *Les triplettes de Belleville*, de Sylvain Chomet.” In Pierre Floquet (ed.) *CinémAction: « CinémAnimationS ».* Paris: Corlet Publications. pp. 97–103; and B. McCann, (2008) “'If it's not Disney, what is it?': traditional animation techniques in Les Triplettes de Belleville”. *French Studies Bulletin* 108: 59–62.

^5^ https://www.imdb.com/title/tt3507676/

^6^Detailed box office figures for this film are available from boxofficemojo.com at https://www.boxofficemojo.com/movies/intl/?page=&wk=2019W26&id=_fDVIZHENIEVVERH01

^7^Carl Schreck, “'More Than Just A Game’: As Doping Disgrace Mounts, Russian Film Revisits Olympic Hoops Glory” Radio Free Europe/Radio Liberty, December 10, 2017, https://www.rferl.org/a/soviet-union-united-states-basketball-final-munich-movie-controversy/28907767.html

^8^Poster available at https://www.alamy.com/stock-photo/soviet-propaganda-poster.html?sortBy=relevant [Accessed 10 June 2023]
